# Multidrug-resistant *Pseudomonas* isolated from water at primary health care centers in Gaza, Palestine: a cross-sectional study

**DOI:** 10.1016/j.ijregi.2025.100671

**Published:** 2025-05-21

**Authors:** Reem Abu-Shomar, Mark Zeitoun, Aula Abbara, Abdelraouf Elmanama

**Affiliations:** 1 Islamic University of Gaza & AL Azhar University, Palestine, Gaza; 2 United Nations University - Institute for Water, Environment and Health, Richmond Hill, Canada; 3 Geneva Water Hub, Geneva, Switzerland; 4 Imperial College, Department of Infectious Diseases, London, UK; 5 Islamic University of Gaza, Palestine, Gaza

**Keywords:** Antimicrobial resistance, Pseudomonas, Water contamination, Conflict, MARI, Gaza

## Abstract

**Objectives:**

To assess the antibiotic resistance profile, multiple antibiotic resistance index (MARI), and genetic determinants of *Pseudomonas* spp. isolated from water sources in primary health care centers in Gaza. This investigation aimed to understand potential risks for nosocomial transmission, particularly in the context of deteriorating healthcare and water infrastructure in conflict zone.

**Methods:**

A total of 64 water samples were collected from five primary health care centers across Gaza (April-August 2022). Isolates were identified using standard microbiological techniques. Antibiotic susceptibility was tested via the Kirby-Bauer disk diffusion method; MARI was calculated, and polymerase chain reaction was used to detect New Delhi metallo-β-lactamase (*NDM*) genes.

**Results:**

*Pseudomonas* spp. were isolated from 59.4% of samples (desalinated: 48.3%; municipal: 68.6%). High resistance was observed to imipenem (84%) and piperacillin (84%), followed by aztreonam (31.6%) and gentamicin (28.9%). The average MARI was 0.4. *NDM* genes were detected in a subset of isolates.

**Conclusions:**

The presence of multidrug-resistant, *NDM*-producing *Pseudomonas* in reverse osmosis and municipal water highlights the urgent need for water safety, screening, and infection control in conflict-affected health care settings.

## Highlights

• Detected resistant *Pseudomonas* in water sources at primary health care centers in Gaza.
• Water systems in health centers may contribute to the nosocomial spread of resistant bacteria.
• Water quality and infection control are vital in health care settings within warzones.
• Water, sanitation, and infection control conditions have likely deteriorated since October 2023.

## Introduction

Antimicrobial resistance (AMR) is a critical global health challenge, threatening the efficacy of antibiotic therapies and contributing to increased morbidity and mortality rates in conflict-affected countries [1]. Multidrug-resistant (MDR) organisms (MDROs), including *Pseudomonas* spp*.*, are increasingly implicated in health care–associated infections, especially in settings with limited infection prevention infrastructure.

In Gaza, the war since October 2023 has caused catastrophic damage to health and water systems, widespread displacement, and a severe humanitarian emergency. Over 90% of Gaza’s population has been displaced, with many seeking refuge in health facilities—spaces that now becoming hotspots for MDRO transmission due to inadequate water, sanitation, and hygiene (WASH) systems. In such environments, contaminated water systems may act not only as reservoirs but also as vectors for resistant pathogens, increasing the likelihood of nosocomial infections in already vulnerable populations.

Healthcare-associated infections caused by MDROs, such as *Pseudomonas* spp., exacerbate these risks, leading to prolonged hospital stays, increased treatment costs, and higher mortality rates [2]. WASH systems are integral to infection prevention in health care settings. However, these systems, if not properly maintained or become contaminated, can contribute to the spread of drug-resistant bacteria. So, instead of preventing infections, the water systems in health care settings can end up being a breeding ground for drug-resistant bacteria, making it easier for them to spread within the health facility.

Water systems in health care facilities, thus, play a dual role in either mitigating or propagating AMR. Inadequately treated water, insufficient chlorination, and biofilm formation within water distribution systems can harbor resistant bacteria, facilitating their transmission through contaminated handwashing water, medical equipment, and surfaces in patient care areas [3]. These factors underscore the need for rigorous water quality management practices to control AMR [4]. Although the global importance of WASH services in health care settings is well-recognized [5], significant gaps remain in understanding the extent of AMR contamination through water sources, particularly, in Gaza’s health care facilities. Given the ongoing destruction of health and water infrastructure, the risk of MDRO exposure through improperly treated water has become even more pressing. As such, research is urgently needed to elucidate the presence of MDR *Pseudomonas* spp., their resistance profiles, and key genetic determinants, e.g. New Delhi metallo-β-lactamase (*NDM)*. Although other non-*Pseudomonas* MDROs (e.g. *Enterobacteriaceae*) are also of concern in waterborne transmission, *Pseudomonas* remains a particularly relevant pathogen due to its intrinsic resistance mechanisms, environmental persistence, and its potential to cause severe infections in health care settings. Addressing these gaps will provide critical evidence for understanding transmission patterns and how best to implement water screening and quality measures in contexts of severe conflict.

This study investigates the prevalence of MDR *Pseudomonas* spp. in water sources in primary health care center (PHC) facilities in Gaza, focusing on reverse osmosis (RO) desalinated and municipal water supplies. The specific objectives were to assess and compare the prevalence of antibiotic-resistant *Pseudomonas* spp. in RO-desalinated and municipal water sources, characterize the antibiotic susceptibility profiles of *Pseudomonas* isolates and calculate the multiple antibiotic resistance index (MARI), and detect the presence of *NDM* resistance genes in these isolates. Although other resistance genes are also important, *NDM* was selected as a marker of high-priority carbapenem resistance for this study due to its strong clinical relevance and the feasibility in conflict-affected settings.

## Methods

### Study design

This cross-sectional study aimed to detect antimicrobial-resistant *Pseudomonas* in water samples from health care facility–based RO desalination systems and municipal water supplies.

### Setting

**Figure 1:**
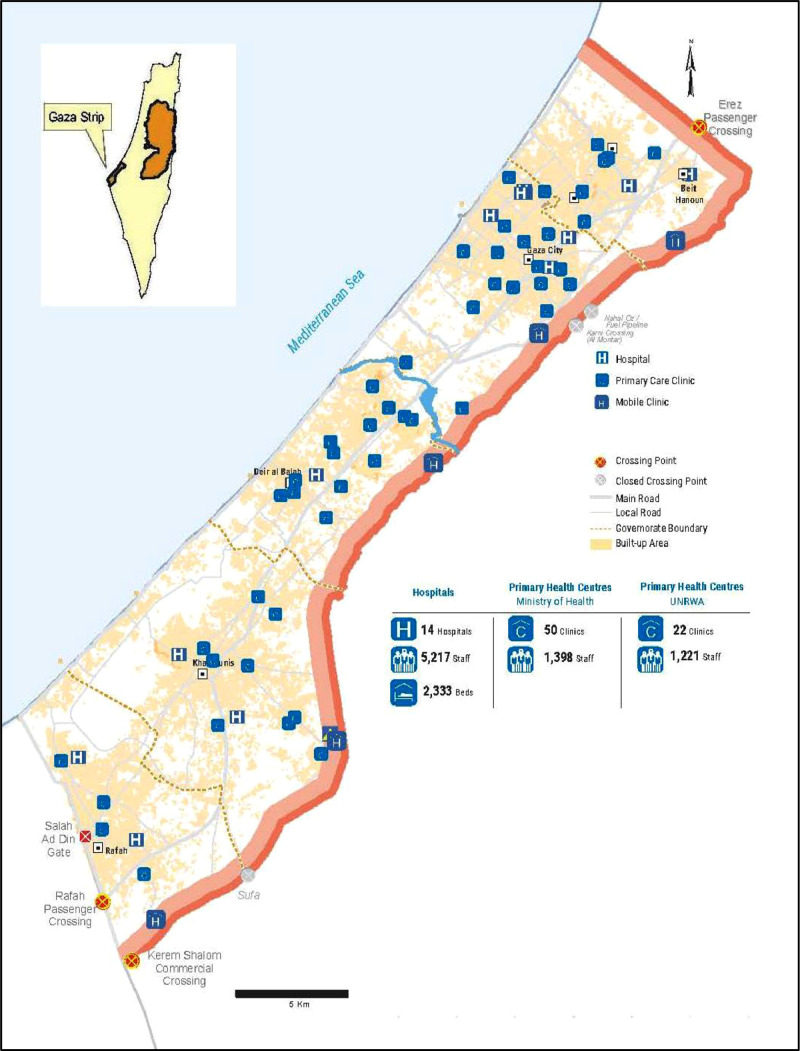
Gaza strip health care facilities map; adopted from [7].

As part of the occupied Palestinian territories, Gaza is a densely populated area, covering a 365-km² stretch along the Mediterranean Sea, with an estimated 2.11 million residents as of 2021 [6]. See Figure 1.

The study was conducted in five out of nine PHCs (level four) managed by the Ministry of Health in Gaza. These centers were selected to represent the five geographic governorates and provide a comprehensive overview of water quality about *Pseudomonas* contamination within health care facilities.

### Ethical considerations

This study did not involve human subjects; however, official authorization was obtained from the Palestinian Ministry of Health to conduct field surveys and collect water samples. All procedures were performed in adherence to ethical guidelines for research involving environmental interventions.

### Data collection

Water samples were collected from two primary sources, municipal water supplies and RO-desalinated water supplies, between April and August 2022. Sampling followed World Health Organization guidelines for water quality monitoring. Sterile 500-ml bottles containing sodium thiosulfate were used to neutralize residual chlorine. Samples were obtained aseptically from taps after 2-3 minutes of flushing, stored at 4-8°C, and processed in the laboratory within 6 hours using standard microbiological techniques for *Pseudomonas* isolation and identification, in line with Clinical and Laboratory Standards Institute guidelines.

Sampling points included handwashing stations, taps, and other water outlets in five selected PHCs. Standard laboratory procedures were used for microbial and genotypic analysis, ensuring consistency and reliability.

### Transport and laboratory analysis

Water samples were transported to the microbiology laboratory at the Islamic University of Gaza within 2 hours of collection. Microbiological analyses were performed using membrane filtration for the enumeration and isolation of *Pseudomonas*. Isolates were identified using the analytical profile index non-fermenters.

### Free residual chlorine and pH tests

Free residual chlorine was measured using the standard diethyl-paraphenylene diamine indicator test. The color intensity was assessed using a portable photometer (Tintometer Group, Germany). Similarly, pH was determined using the phenol red method, and the color was assessed with the same portable photometer.

### Antimicrobial susceptibility testing

Antimicrobial susceptibility testing was conducted using the Kirby-Bauer disk diffusion method following Clinical and Laboratory Standards Institute guidelines [7]. MARI was calculated as the ratio between the number of antimicrobials that an isolate is resistant to and the total number of antimicrobials the organism tested against.

### Molecular detection of resistance genes

Polymerase chain reaction was applied to detect *NDM* resistance genes in *Pseudomonas* isolates using the primers indicated in Table 1.

**Table 1:** Primers of antimicrobial resistance genes.

Name	Sequence^a^	Annealing temperature^b^
*NDM* F	5′-GGTTTGGCGATCTGGTTTTC-3′	58
*NDM R*	5′-CGGAATGGCTCATCACGATC-3′	60

a Reference [9].

b Annealing temperature (°C) (Metabion International AG, Germany).

### Data analysis

Data were analyzed using SPSS software (version 23). Descriptive statistics, including frequencies and percentages, summarized the prevalence and resistance profiles of *Pseudomonas* isolates. Inferential statistics, such as chi-square tests and *t* tests, were used to assess associations between variables, with significance determined at a *P*-value threshold of <0.05.

## Results

A total of 64 water samples were collected from primary health care facilities in Gaza. These comprised 29 of 64 (45%) from desalinated water and 35 of 64 (55%) from municipal (non-desalinated) water sources.

### Microbial identification and enumeration

**Figure 2:**
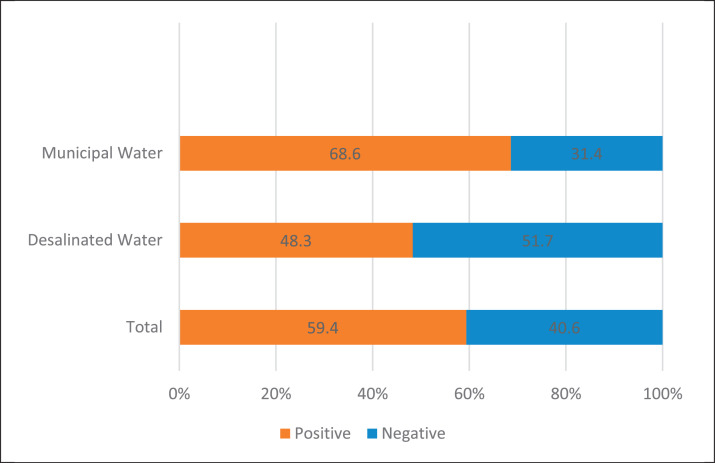
Presence of *Pseudomonas spp.* by water supply type.

Microbial growth of *Pseudomonas* spp*.* on cetrimide media was observed and confirmed by biochemical tests in 59.4% (38 of 64) of water samples. Within water samples, *Pseudomonas* spp*.* was detected in 48.3% (14 of 29) of desalinated and 68.6% (24 of 35) of municipal water, with no statistically significant difference (*P* = 0.16). See Figure 2.

As indicated in Table 2, *Pseudomonas* spp. were enumerated as follows: 5.3% of samples had 1-10 CFU/ml, 57.9% had 11-100 CFU/ml, and 36.8% exceeded 100 CFU/ml, indicating high bacterial count in more than a third of isolated samples.

**Table 2:** *Pseudomonas* count on cetrimide media.

*Pseudomonas* count (CFU/100 ml)	n	%
**1-10**	2	5.3
**11-100**	22	57.9
**>100**	14	36.8

### Free residual chlorine and pH levels

The analysis of residual chlorine levels revealed that 79.7% of the samples contained no detectable chlorine. The average residual chlorine concentration was 0.03 mg/l. The average pH of water samples was 7.2, and although nearly all samples fell within permissible pH ranges, desalinated water had significantly lower pH values than municipal sources (*P* = 0.006). See Table 3a.

**Table 3a:** Average free residual chlorine and pH per water supply type.

	Water supply	n	Mean	SD	T	*P*
Free residual chlorine (mg/l)	Desalinated	29	0.028	0.0528	−0.062	0.621
	Municipal	35	0.029	0.0710		
pH test	Desalinated	29	6.7	0.1575	−19.05	0.006
	Municipal	35	7.6	0.2292		

Considering the general low chlorination levels in water samples, the average free residual chlorine was slightly higher in water samples that tested negative for *Pseudomonas* (0.035 mg/l) than samples that tested positive (0.024 mg/l). As shown in Table 3b, the variation in the average free residual chlorine was statistically insignificant (*P* = 0.49).

**Table 3b:** Average FRC per *Pseudomonas* growth.

	Growth	n	FRC Mean	SD	T	*P*
FRC (mg/l)	Positive	38	0.02	0.07	0.69	0.49
	Negative	26	0.04	0.06		

FRC, free residual chlorine.

### Antibiotic susceptibility

As presented in Table 4a, high resistance rates were observed for imipenem (84%), piperacillin (84%), aztreonam (31.6%), gentamicin (28.9%), and ceftazidime-avibactam (32.7%). No significant differences were detected in resistance rates between isolates from desalinated and municipal water samples.

**Table 4a:** Antibiotic resistance profile for *Pseudomonas* by sample type (n = 38).

	Water supply								*P*	Total (n = 38)			
	Desalinated (n = 14)				Municipal (n = 24)								
	Sensitive		Resistant		Sensitive		Resistant			Sensitive		Resistant	
	N	%	n	%	N	%	n	%		n	%	n	**%**
Aztreonam	10	71.4	4	28.6	16	66.7	8	33.3	0.515	26	68.4	12	31.6
Cefepime	13	92.9	1	7.1	20	83.3	4	16.7	0.446	33	86.8	5	13.2
Ceftazidime	13	92.9	1	7.1	21	87.5	3	12.5	0.653	34	89.5	4	10.5
Ceftazidime-avibactam	11	78.6	3	21.4	18	75.0	6	25.0	0.896	29	76.3	9	23.7
Gentamicin	9	64.3	5	35.7	18	75.0	6	25.0	0.392	27	71.1	11	28.9
Imipenem	3	21.4	11	78.6	3	12.5	21	87.5	0.806	6	15.8	32	84.2
Meropenem	13	92.9	1	7.1	19	79.2	5	20.8	0.301	32	84.2	6	15.8
Piperacillin	2	14.3	12	85.7	4	16.7	20	83.3	0.920	6	15.8	32	84.2

^a^significant at *p* <0.05.

### Multiple antibiotic resistance index

The MARI for *Pseudomonas* spp. was calculated to be 0.4, indicating a high-risk source of antibiotic resistance exposure. See Table 4b.

**Table 4b:** Multiple antibiotic resistance index.

Multiple antibiotic resistance index	n	%
0.0	1	2.6
0.1	5	13.1
0.2	-	-
0.3	9	23.7
0.4	10	26.3
0.5	8	21.1
0.6	2	5.3
0.7	-	-
0.8	1	2.6
0.9	2	5.3
**Average**		**0.4**

### Molecular testing for antimicrobial resistance genes

Polymerase chain reaction analysis identified the carbapenem-resistant gene *NDM* in 26.3% (10 of 38) of *Pseudomonas* spp. isolates. This included five of 14 (35.7%) isolates from desalinated water samples and five of 24 (20.8%) isolates from municipal water samples. Table 4c shows the *NDM* distribution among *Pseudomonas* spp. isolates in desalinated and municipal water samples.

**Table 4c:** New Delhi metallo-β-lactamase by water supply type.

	*New Delhi metallo-β-lactamase*	
	n	%
**Desalinated (n = 14)**	5	35.7
**Municipal (n = 24)**	5	20.8

## Discussion

The findings from this study highlight the significant presence of MDR *Pseudomonas* spp. in municipal and desalinated water supplies in Gaza’s PHCs, indicating a potential serious public health risk. The detection of *Pseudomonas* in 59.4% of water samples and the identification of the *NDM*-resistant gene in 26% of *Pseudomonas* isolates underscore the urgent need for effective water safety and AMR monitoring within health care facilities. These contamination levels exceed national standards and World Health Organization guidelines [8], emphasizing a widespread issue of waterborne AMR in Gaza in health care settings.

The study reveals concerning high levels of bacterial contamination with *Pseudomonas* spp., suggesting that poor water quality may contribute to the transmission of AMR pathogens, complicating clinical outcomes and increasing the risk of outbreaks. Several studies around the world reported the effect of *Pseudomonas* in health care water systems and water-dependent equipment, which can result from external contamination or from existing biofilms that develop within the water system. It can also be transmitted from patients to the water system through hand washing and other activities [9], posing a risk of transmission to patients. This has become more urgent in Gaza, where the war since December 2023 has led to widespread fatalities, injuries, destruction of health facilities, lack of water, forced displacement, and civilians seeking safety in health facilities including in PHCs. In addition, an estimated 113,000 patients have been wounded, many of whom have undergone surgical interventions involving prosthetic implants having prosthetic material in place [10,11], A recent study on fracture-related infections during medical missions to Gaza reported that 30% of patients with war-related fractures developed suspected infections, predominantly after explosive injuries. The high burden of fracture-related infections was attributed to malnutrition, inadequate sanitation, limited sterile supplies, and the absence of standard infection control practices—conditions that mirror the broader health care infrastructure challenges in conflict zones. These findings underscore the urgent need for comprehensive infection prevention strategies, particularly, in settings such as Gaza, where patients with wounds or prosthetic materials are highly susceptible to infections—such as those caused by *Pseudomonas*—from environmental sources, including contaminated water systems [12], something that can be associated with biofilm formation.

This study demonstrated that desalinated and municipal water can serve as a potential source of infection with AMR MDR *Pseudomonas* considering the high average of MARI (0.4). Conversely, the reported contamination of a health care facility’s plumbing system with *Pseudomonas* in France shows that isolates were fully susceptible to antibiotics such as aztreonam, cefepime, ceftazidime, imipenem, meropenem, and piperacillin [13]. In Tanzania, the isolated *Pseudomonas* from water samples at health care facilities were resistant to aztreonam (100%), gentamicin (12.8%), and piperacillin (18%) [14]. Furthermore, *Pseudomonas* was the most commonly isolated bacteria from tap water and water used for hemodialysis and bronchoscope flushing in Italy. The isolates exhibited variable patterns of AMR [15]. The wide variations in resistance patterns among various studies could be attributed to the levels of antimicrobial use, the presence or absence of laws and regulations regarding antimicrobial use in various sectors, general resistance rates among clinical and environmental isolates, and many other possible reasons. However, there is a wide agreement that MDR phenomena complicate treatment options and contribute to the spread of resistant infections, making infection control more challenging [4].

In this study, 79% of *Pseudomonas* isolates in desalinated water and 88% in municipal water was were resistant to imipenem. This is much higher than a previous study (12.1%) that was conducted on clinical and environmental samples collected from hospitals in the Gaza Strip [16]. Although carbapenem-resistant *Pseudomonas* spp. are widely reported worldwide, including in clinical isolates from Lebanon [17], more relevant comparisons can be drawn from studies that explore the presence of *Pseudomonas* spp. in environmental water sources across the region. A recent study investigated the chemical and microbiological quality of drinking water across five Lebanese governorates and found widespread contamination across all water sources, including station-filtered, tap, and artesian well water. Notably, *Pseudomonas aeruginosa* was among the bacterial species detected, highlighting the potential for waterborne transmission and the importance of monitoring such pathogens in non-clinical environments [18]. These findings reinforce the significance of our investigation in Gaza, where conflict has further compromised water infrastructure and where contaminated healthcare water supplies may serve as a reservoir for MDR pathogens. In the Kingdom of Saudi Arabia, imipenem resistance was found in 38.6% of *Pseudomonas* isolates [19]. The genotyping results of the study indicated that 26% (35.7% in desalinated and 20.8% in municipal water) of *Pseudomonas* isolates possess the *NDM*-resistant gene.

Desalinated and municipal water sources were found to harbor MDR *Pseudomonas* bacteria. Although desalinated water showed a slightly lower level of bacterial growth, it did not completely mitigate the risk of MDR transmission. This indicates that water safety issues, such as cross-contamination during distribution and storage, coupled with inadequate free residual chlorine levels, are significant contributing factors to the persistence of MDR *Pseudomonas* in these water supplies.

The presence of MDR *Pseudomonas* in health care facility water supplies can lead to severe health care–associated infections, particularly, among vulnerable populations such as patients with injuries and immunocompromised individuals. These findings underscore the critical need for routine testing of *Pseudomonas* spp. in water systems, which has not been part of standard safety protocols in Gaza’s health care facilities. This study highlights the urgency of targeted interventions to restore sustainable health and WASH services in Gaza, improve water safety, enhance infection control practices, and monitor MDR to prevent the further spread of resistant strains, an issue that is even more critical in war zones and areas of devastation.

### Strengths and limitations

This is one of the few studies that screen water sources in PHCs in Gaza and, to the best of our knowledge, the only study to characterize the presence and extent of drug-resistant *Pseudomonas* spp. in health care facilities in Gaza. Limitations include the relatively small sample size, although the number of samples taken is comparable to similar studies in other countries. In addition, this study was conducted in 2022, before October 7, 2023, which devastated health and microbiology facilities across Gaza. However, the data from this study remains valuable for humanitarian and health care workers seeking to understand the presence of MDR *Pseudomonas* spp. in Gaza’s health care facilities.

## Conclusion

The detection of MDR *Pseudomonas* spp., including *NDM*-positive strains, in municipal and desalinated water sources in PHCs in Gaza raises serious concerns about water safety and infection control, particularly, amid conflict. Inadequate disinfection, indicated by low free residual chlorine levels, likely contributes to bacterial persistence and potential nosocomial transmission, especially among wounded and vulnerable patients. The presence of *Pseudomonas* in desalinated water suggests possible post-treatment contamination or biofilm formation in distribution systems.

These findings highlight the urgent need to implement routine water quality screening and maintenance protocols, even in conflict settings where such measures are logistically challenging. Targeted interventions—such as improved chlorination, system disinfection, and point-of-use treatments—should be integrated with broader AMR control strategies, including antimicrobial stewardship and infection prevention and control. In war-affected regions such as Gaza, protecting health care water systems is essential to mitigating AMR risks and ensuring safer environments for patient care.

## Declarations of competing interest

The authors have no competing interests to declare.
